# COMPARATIVE ANALYSIS OF TISSULAR RESPONSE AFTER ABDOMINAL WALL REPAIR USING POLYPROPYLENE MESH AND BOVINE PERICARDIUM MESH

**DOI:** 10.1590/0102-672020200003e1527

**Published:** 2022-01-05

**Authors:** Marina BAKRI, Fernanda Christo LOVATO, Géssica de Mattos DIOSTI, Yorgos Luiz Santos de Graça SALLES, Paulo Henrique Brites MOREIRA, Luiz Martins COLLAÇO, Nicolau Gregori CZECZKO, Osvaldo MALAFAIA, Luiz Fernando KUBRUSLY

**Affiliations:** 1 Institute for Medical Research, Curitiba, PR, Brazil; 2 Mackenzie Evangelical Faculty of Paraná, Curitiba, PR, Brazil

**Keywords:** Bovine pericardium, Polypropylene, Inflammatory process, Pericárdio bovino, Polipropileno, Processo inflamatório

## Abstract

**Background:**

The use of polypropylene meshes for surgical repair of the abdominal wall contributes to a reduction of the of recurrence rates of hernias or defects. However, its intra-abdominal use comes along with the formation of adhesions and several complications. The study and the search for alternative materials, including bovine pericardium, have been regarded as an option for the correction and treatment of resulting hernias with better adaptations and effectiveness.

**Aim:**

Evaluating the inflammatory process of the bovine pericardium in comparison with the inflammatory process of synthetic polypropylene mesh.

**Method:**

Bovine pericardium mesh and polypropylene mesh were placed, both on the same animal. The first group had the mesh removed for analysis on day 20, and the second group on day 40. The variables congestion, granulation, giant cells, necrosis, acute inflammation, chronic inflammation and collagen were analyzed.

**Results:**

All variables were found in greater numbers as a response to the polypropylene mesh, except for the collagen, which, on day 40, was greater in response to the bovine pericardium mesh.

**Conclusion:**

The data in this study suggest that there is less inflammatory reaction in response to bovine pericardium mesh when compared to polypropylene mesh.

## RESUMO

**Racional:**

O uso de telas de polipropileno para a correção cirúrgica da parede abdominal contribui para redução dos índices de recidiva das hérnias ou defeitos. No entanto, o seu uso intra-abdominal cursa com a formação de aderências e diversas complicações. O estudo e a busca por materiais alternativos, como pericárdio bovino, têm se mostrado uma opção na correção e tratamento de hérnias que resultem com melhores adaptações e efetividades.

**Objetivo:**

Avaliar o processo inflamatório do pericárdio bovino em comparação ao processo inflamatório da tela sintética de polipropileno.

**Método:**

Foi realizada a colocação de tela de pericárdio bovino e polipropileno, ambas no mesmo animal. O primeiro grupo as teve retiradas para análise no dia 20, e o segundo grupo no dia 40. Foram analisadas as variáveis congestão, granulação, células gigantes, necrose, inflamação aguda, inflamação crônica e colágeno.

**Resultados:**

Todas as variáveis foram encontradas em maior número como resposta a tela de polipropileno, exceto a variável colágeno, que no dia 40 apresentou-se em maior quantidade em resposta à tela de pericárdio bovino.

**Conclusão:**

Há menor reação inflamatória em resposta a tela de pericárdio bovino, quando comparada com a de polipropileno.

## INTRODUCTION

Surgical repair or reinforcement of the tissues that compose the wall of the abdominal cavity is one of the most frequent needs in human surgery^12^, due to both the scarcity of tissues and the high tension in the suture line, which results in the occurrence of high rates of incisional hernia^8^.

The use of meshes for surgical repair of hernia defects of the abdominal wall contributes to reducing the recurrence^11^ ^,^ ^17^. However, its intra-abdominal use comes along with the formation of adhesions and several complications^2^.

The first reported use of tissue for hernioplasty is dated 1958 by Usher et al^16^, who used a polypropylene mesh. This remains the most used material^18^ because it is safe and offers several advantages in its use, including its availability, easy handling, low cost and good tolerance^9^.

Polypropylene is a synthetic material that produces little tissue reaction and good tensile strength, which strength lasts for several years after its use in living organisms. However, even with this possible choice, the use of meshes still has several side effects^4^, such as increased postoperative pain, abdominal adhesions and areas of fibrosis and increased risk of infection due to the placement of the prosthesis^18^.

Since the 1960s, biological membranes as implant material for the repair of organs and tissues have been used in Brazil. The advantages of using this type of material include the ease in obtaining, its low cost, simple preparation, viable sterilization, easy storage, little or no tissue reaction, and a long period of viability as an implant. Bovine pericardium is one of the most used biological membranes, being composed almost exclusively of collagen. Due to this characteristic, bovine pericardium easily adapts to the different situations to which it is submitted in the surgical practice^5^. 

Although reports in the literature indicate that bovine pericardium has a greater tensile strength in relation to certain synthetic meshes, calcifications and immune rejection responses should be considered, taking into account the indications in the use of this material^13^. The bovine pericardium is the bovine heart surrounding membrane, being a biological tissue widely studied in the literature to produce products of medical use, many of which already in clinical use. The structure of this biological tissue basically comprises collagen and it is used in medicine after being physically and/or chemically treated for improving its mechanical and immunogenic properties and controlling its degradation or calcification. It is used for example in the production of cardiac and vascular prostheses, repair of ligaments, controlled drug delivery systems, hemostatic agents, grafts and others. Valve prostheses made with bovine pericardium have a good hydrodynamic performance and low thrombogenicity^3^.

Since the beginning of the last century, the use of prostheses has become constant in abdominal hernia surgery, especially for inguinal hernia. The study and search for alternative materials, such as bovine pericardium, have proved to be an option in the correction and treatment of hernias resulting in better adaptations and effectiveness^7^.

The objective of this study is to evaluate the inflammatory process and formation of collagen fibers in response to bovine pericardium compared to the inflammatory process and formation of collagen fibers in response to the synthetic polypropylene mesh.

## METHODS

This study has a qualitative and quantitative experimental character, and therefore, the abdominal wall defect induction and subsequent correction with polypropylene mesh and bovine pericardium mesh was performed. It was approved under CEP number 1513/2016. The procedures with the animals complied with that recommended by the Ethics Committee on the Use of Animals (CEUA) of Evangelical Faculty of Paraná (FEPAR), Curitiba, PR, Brazil.

A total of 19 male guinea pigs, weighing between 300 and 400 g, were collected from the Paraná Institute of Technology (TECPAR) vivarium. The animals were kept during the experiment at IPEM’s vivarium, in 47x34x18 cm plastic boxes, with two animals each, lined with shavings, in 12-h light/dark cycle (light from 7am to 7pm) at a temperature of 22±2º C. The animals were treated daily with filtered water and appropriate ration fed freely.

The model used was abdominal wall defect correction using mesh, divided into two groups of nine guinea pigs in group 1 and 10 guinea pigs in group 2. In group 1 surgical correction using polypropylene synthetic mesh and bovine pericardium biological tissue with tissue removal and animal death after 20 days was done. In group 2 the same procedure was performed, but the animal death was after 40 days.

**FIGURE 1:**
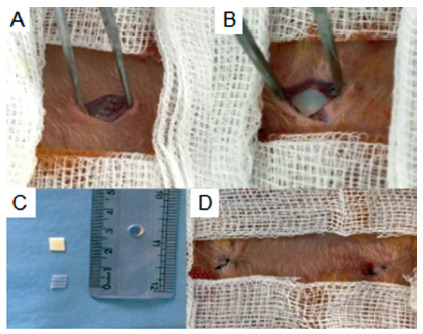
Meshes inserted into the abdominal wall: A) Left side with polypropylene mesh; B) right side with bovine pericardium mesh; C) cut meshes; D) after abdominal closure.

A mixture of xylazine hydrochloride 5 mg and ketamine hydrochloride 40 mg per body weight in kilograms intraperitoneally was used to anesthetize the animals, and they were positioned for surgery in the dorsal decubitus position. The surgical intervention began with trichotomy and disinfection of the abdominal region, followed by an incision to create a 1 cm defect in the abdominal wall through the epithelium to the peritoneal tissue, for subsequent correction with polypropylene mesh on the right side and bovine pericardium mesh on the left side (Figure 1).

The meshes were 0.5 x 0.5 cm and were accommodated in the abdominal cavity above the peritoneum.

The abdominal cavity was closed with separate stitches in the cutaneous plane, and the abdominal muscle layer was closed with continuous suture.

Tramadol was used for postoperatively analgesia at a dose of 5 mg per kg every 12 h for four days and we also used amoxicillin at 20 mg per every 48 h for four days. The surgical wound was cleaned with iodine. For animal welfare concern, knowing that the environment influences the behavioral and physiological state of animals, we implemented environmental enrichment measures including: appropriate environment, feed, temperature, space and PVC pipe in the cages.

### Death of the animals and tissue resection for histopathological analysis

**FIGURE 2:**
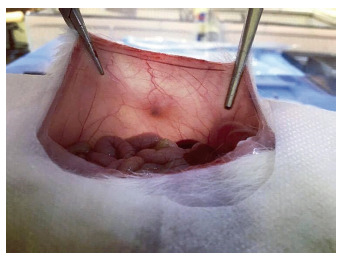
Mesh analysis by transparency

After the median abdominal incision, the meshes were found by transparency, so that the specimen was removed from the correct place (Figure 2).

The surgical specimen was removed in a single block and immediately immersed in 10% buffered neutral formaldehyde, remaining in the fixer for 72 h at room temperature.

The material was cut in 6-micrometer sections and stained using hematoxylin-eosin (H&E) technique for the cellular and capillary elements, and the Mallory’s trichrome technique for fibrous collagen fibers. The analysis was made using an Olympus^®^ optical microscope, model DX 50 to evaluate the inflammatory process of each material, i.e.: congestion, formation of granulation tissue, presence of acute inflammation, presence of chronic inflammation, presence of giant cells, and necrosis. 

The inflammatory cells analyzed were histiocytes, neutrophils, lymphocytes, giant cells and calcified cells. The histiocyte considered in this histopathological study as a type of macrophage of endothelial reticulum origin, is typically immobile and inactive but, when stimulated, it can become active; the neutrophil, a polynuclear leukocyte with neutrophilic granulations; the lymphocyte, a type of leukocyte ranging between 10-12 microns in diameter, with round nucleus with condensed chromatin and scarce basophilic cytoplasm; the giant cell, as that formed by the union of several distinct cells. Regarding the presence of fibrosis, it was classified as absent, scarce and focal, diffuse and moderate or diffuse and intense deposition^6^.

On the last day of the experiment, day 20 for the first group and day 40 for the second, anesthesia was applied for the death of the animals. The anesthetic technique involved intraperitoneal administration using ketamine hydrochloride at the dose of 120 mg per kg of the animal and xylazine hydrochloride at a dose of 15 mg per kg of the animal.

### Histological evaluation

Two staining colors were used for the histopathological analysis: H&E, according to Yasojima et al^18^, and Mallory’s trichrome. A pathologist without previous knowledge of the division of the groups received a slide for each animal and evaluated the pattern of histological changes. In H&E staining the parameters of congestion, granulation, giant cells and necrosis were analyzed and classified as S=presence or N=absence. Acute and chronic inflammation are classified as 0=absence, 1=slight, 2=moderate, and 3=intense.

The Mallory’s trichrome quantified the arrangement of collagen fibers, being classified as 0=absence, 1=scarce and focal deposition, 2=moderate and diffuse deposition, and 3=intense and diffuse deposition.

### Statistical analysis

The results of H&E and collagen fiber variables were described by frequencies and percentages. Fisher’s exact test was used to compare the groups defined by the day of death of the animals (20 days or 40 days) in relation to the variables evaluated. The comparisons between the two types of meshes (polypropylene or bovine pericardium) were made using the binomial test. Values of p<0.05 indicated statistical significance. The data was analyzed using the IBM SPSS Statistics v.20 software.

## RESULTS

The analysis was performed based on the data of nine animals of the group submitted to euthanasia after 20 days and 10 after 40 days. Both types of mesh (polypropylene and bovine pericardium) were used in each animal. For the comparative analysis between the types of screen (in the same animal) were included the cases that had evaluation of the variable in the two types of mesh. The “necrosis” variable was not analyzed, since all animals in the experiment had no characteristic of this characteristic.

**FIGURE 3:**
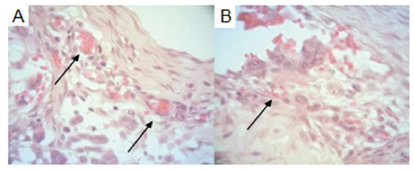
Photomicrographs evidencing alterations in the abdominal wall (400x H&E): A) connective tissue with congested vessels (arrows); B) granulation tissue (arrow).

In relation to congestion, there was presence of alteration in 66.7% of polypropylene tissues in group 1 and 60% in group 2 (p>0.05). In the group of tissues with bovine pericardium, there was congestion in 33.3% in group 1 and 10% in group 2 (p>0.05). Although without significant difference, the granulation variable was present in 100% of polypropylene mesh materials in group 1 and in 55.6% with bovine pericardium mesh. In group 2 this variable was present in 80% with polypropylene and only 20% in the pieces with bovine pericardium (Figure 3).

**FIGURE 4:**
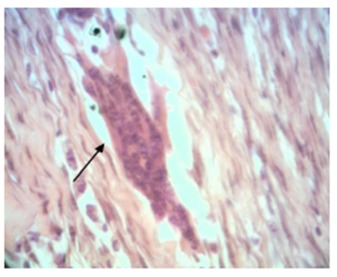
Photomicrograph demonstrating giant cell (400x H&E - arrow)

The presence of giant cells was evidenced in 66.7% of the analyzed pieces with polypropylene of group 1 and in 33.3% of the pieces with bovine pericardium. In group 2 this variable was present in 60% of the pieces with polypropylene and in no part with bovine pericardium (both with p>0.05, Figure 4).

Regarding acute inflammation, no alteration was found in any part of the first group and did not present a statistically significant difference.

**FIGURE 5:**
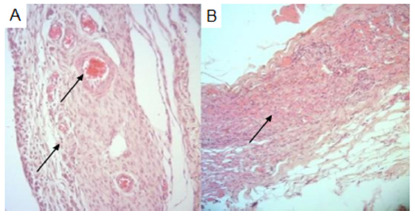
Photomicrographs evidencing changes of chronic inflammatory process (A - arrows) and minimal scar area (B - arrow) - 100x H&E

In relation to chronic inflammation, there were alterations in 66.7% of the pieces with polypropylene of group 1 and in 22.2% of the pieces with bovine pericardium of group 1, with p>0.05. However, in group 2, a significant difference (p=0.004) was found when compared to the presence of chronic inflammation in parts with polypropylene (90%) and bovine pericardium (0%, Figure 5).

**FIGURE 6:**
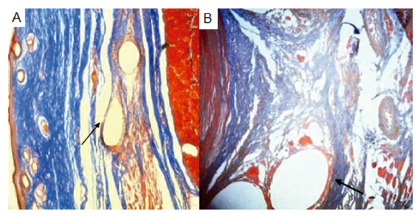
Photomicrographs showing changes to Mallory’s trichrome: A) photomicrograph demonstrating on the arrow cuts of skin with presence of polypropylene and slight deposition of collagen around (Mallory 50x); B) photomicrograph demonstrating on the arrow area cicatricial with remnants of the polypropylene mesh and moderate degree of fibrosis around (Mallory 400x)

The collagen fibers were found in 66.7% of the pieces with polypropylene and in 33.3% of the pieces with bovine pericardium in group 1 (p>0.05). In group 2, the variable was found in 62.5% of the pieces with polypropylene and 87.5% of the pieces with bovine pericardium (p>0.05, Figure 6).

## DISCUSSION

The present study aims to analyze the healing of the abdominal wall tissues after the use of two meshes, so that we can compare the tissue response. The subject was chosen due to the high number of surgeries performed with meshes. A guinea pig was used for each two implants (bovine pericardium mesh and polypropylene mesh) to reduce the number of animals in the experiment. Caviaporcellus was chosen because its size and docile nature facilitate the manipulation of the abdominal tissues.

When the tissue responses of the pericardium meshes are compared to each other on day 20 and day 40, they show a decrease in the prevalence of the variables granulation, congestion, giant cells and chronic inflammation, showing better results compared to polypropylene shares when compared to each other. This demonstrates that these factors can be reduced as the days pass when using the bovine pericardium biological mesh.

In the next step of the analyzes the variables for one mesh were compared to the other. When the results obtained by the death of the animals on day 20 are compared, we noticed that while 66.7% of the specimens with polypropylene mesh showed congestion, which figure decreased to 60 on day 40, for the pericardium mesh this value decreased from 33.3% to 10.0%. This decrease also occurred in relation to the variables giant cells and granulation, both resulting in p=0.031. This shows us the ability of the tissue surrounding the implant to adapt more quickly when in response to the biological tissue.

The variable acute inflammation is also improved when using the bovine pericardium patch. For the variable chronic inflammation, the polypropylene group presented the variable in 66.7% on day 20, while the pericardium group only had 22.2%. The analysis of the same variable at day 40 show otherwise that 90% of the polypropylene meshes caused chronic inflammation, while no bovine pericardium mesh presented this variable. This brings us a p=0.004, corroborating with studies^15^ which characterize the synthetic screens as more inflammatory. It also shows the importance of a biological tissue in the tissue response, as already observed by Abouelnasr, K. S. et al^1^, and in studies related to the area of cardiac surgery.

Analyzing the variable collagen, while on day 20 the polypropylene mesh group presented the variable in 66.7%, only 33.3% was present in the pericardium group. However, on day 40, we noticed that while in the polypropylene group the variable was present in 62.5%, in the bovine pericardium group it was present in 87.5%. It is noted that there was a 54.2% increase in the presence of collagen in the bovine pericardium group.

Analyzing the results of the present study we note that although for all variables the polypropylene meshes resulted in increased tissue response and greater inflammation, the bovine pericardium meshes resulted in longer production of collagen for a longer term. The value of p=0.004 demonstrates the presence of much greater chronic inflation in response to polypropylene and corroborates with studies that show that synthetic meshes of this material have inflammation as a major side effect, generating an exacerbated response of the tissue, and may present adhesions and other complications^14^. On the other hand, when we analyze the variable collagen, we can assume that the increased collagen in the long term suggests that, although the bovine pericardium meshes do not cause intense local inflammation, they can form collagen that brings consistency and tensile strength to the abdominal wall after a defect repair surgery, as noted by Ricciardi, Bruno Filippi *et al* ^10^, who showed the best tissue response and least adhesion in response to a collagen-enveloped mesh when compared to a collagen-free mesh.

## CONCLUSION

The data of this study suggests that there is less inflammatory reaction and increased formation of collagen fibers in response to bovine pericardium mesh when compared to the polypropylene mesh.
